# Modeling conformational transitions in kinases by molecular dynamics simulations: achievements, difficulties, and open challenges

**DOI:** 10.3389/fgene.2014.00128

**Published:** 2014-05-13

**Authors:** Marco D'Abramo, Neva Besker, Giovanni Chillemi, Alessandro Grottesi

**Affiliations:** ^1^CINECARome, Italy; ^2^Dipartimento di Chimica, Sapienza University of RomeRome, Italy; ^3^Dipartimento di Scienze e Tecnologie Chimiche, Università di Roma “Tor Vergata,”Rome, Italy

**Keywords:** molecular dynamics, conformational transitions, kinases, computational modeling, conformational pathways

## Abstract

Protein kinases work because their flexibility allows to continuously switch from inactive to active form. Despite the large number of structures experimentally determined in such states, the mechanism of their conformational transitions as well as the transition pathways are not easily to capture. In this regard, computational methods can help to shed light on such an issue. However, due to the intrinsic sampling limitations, much efforts have been done to model in a realistic way the conformational changes occurring in protein kinases. In this review we will address the principal biological achievements and structural aspects in studying kinases conformational transitions and will focus on the main challenges related to computational approaches such as molecular modeling and MD simulations.

## Introduction

Protein kinases, one of the largest gene families in eukaryotes, are a class of enzymes which regulates several key cellular processes, such as cell growth and differentiation.

They all achieve their function by favoring particular structural arrangements, able to catalyze protein phosphorylation. Despite the high degree of the conservation of the overall fold, each kinase is able to respond to specific signals tuning its activity.

Therefore, these molecules represent a unique example of the nature's ability to introduce diversification through relatively small variations to a highly conserved motif.

Such variations, which rarely modify the optimized structure on the protein free-energy minimum, can severely affect protein flexibility which in turn might modify the transition patterns designed to be optimal for their function.

Protein kinases probably represent one of the most remarkable examples of the importance of the protein flexibility: in fact, they regulate—continuously switching from active to inactive state—multiple biological processes by posttranslational phosphorylation of serine, threonine and tyrosine residues. In this context, the knowledge of their structures, although mandatory, it is not sufficient for a complete understanding of their function, thus, the description of their conformational transition pathways is of paramount importance.

However, the experimental techniques able to accurately describe the transient/intermediate states populated between the conformational changes are still under development (Ban et al., 2011; Waldrop, 2014), thus, computational methods can greatly help in this case.

Atomistic Molecular Dynamics (MD) simulations—based on the classical mechanics laws and using well-refined empirical force fields—are probably the most accurate computational technique for the study of protein flexibility, being able to describe the dynamical evolution of molecular systems at atomistic level of detail. Unfortunately, the time-scale reached by standard MD simulations is still limited by the computational power to the microsecond, thus excluding processes occurring on longer times, such as the conformational transitions of almost all kinases. Therefore, several theoretical-computational techniques able to enhance the conformational sampling were applied to model such a process.

Here, after presenting the structural features of protein kinases, we discuss some recent results obtained in modeling kinase conformational transitions by enhancing sampling techniques and brute-force approaches, highlighting their advantages and limits. Finally, due to the involvement of this protein family in very important diseases such as cancer, we briefly introduce the results reached in drug design using computational techniques able to provide a complete description of the accessible conformational space. We discuss their still full unexploited potential to further enhance the kinase inhibitor performance.

## Kinase structure and conformational variability

**Figure 1:**
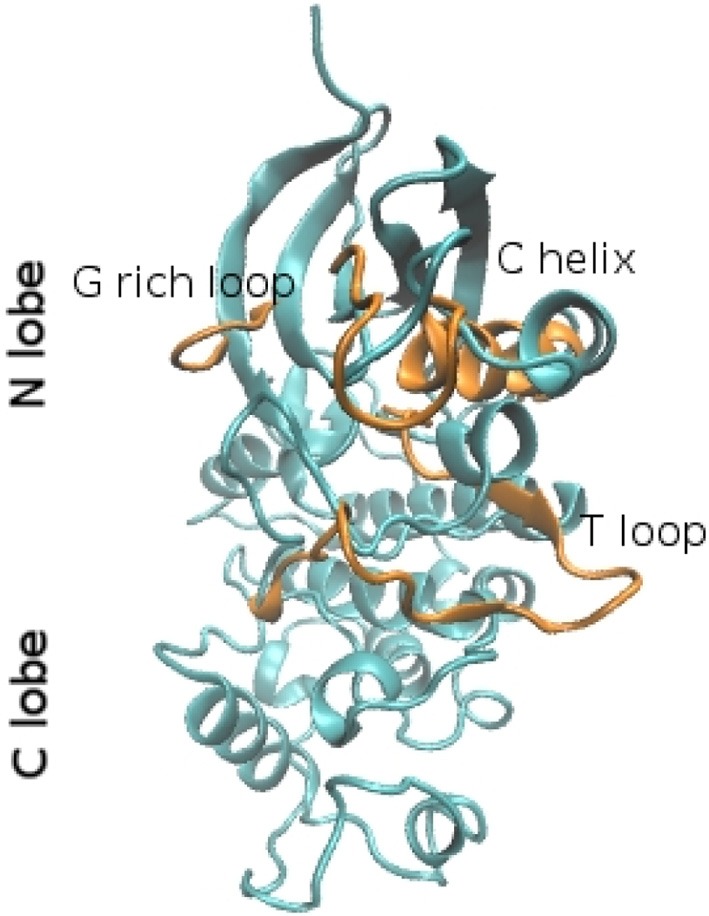
**Ribbon representation of the molecular fold of the CDK2 in the closed (cyan) and open conformation (orange)**. The structures were extracted from molecular dynamics trajectories (Bešker et al., 2013).

To get insights into the structure/dynamics/function relationship we need to describe their molecular structure in detail. Although the number of human protein kinase family members is large, the existing X-ray crystallographic studies showed that the three-dimensional (3D) structures of their catalytic domains are similar. Studies based on X-ray structures of PKA revealed that catalytic subunits of protein kinases share a conserved core consisting of two lobes. The N-terminal small lobe (N-lobe) and C-terminal large lobe (C-lobe). These two lobes host a deep pocket that accommodates a molecule of ATP bound to one or two divalent cations: magnesium or manganese that compensate for the strong negative charge of the ATP phosphates (Kornev and Taylor, 2010). This special structural arrangement is required because the phosphoryl transfer reactions are highly depend on the bond distances and charge distribution in the reaction transition state. In particular, structural studies performed with transition state mimics showed that the presence of Mg2+ ions and positively charged groups in its vicinity would imply an associative mechanism for phosphoryl transfer, thus optimizing the overall reaction (Madhusudan et al., 2002). The N-lobe usually includes five β-strands and an α-helix (hereafter called αC-helix). Despite the fact that the β-strands form a relatively rigid antiparallel β sheet, the N-lobe is very dynamic and flexible. The C-lobe is mainly α-helical (Figure 1) and includes the activation segment, a 20–35 residues stretch located between a conserved DFG motif, and the APE motif (Huse and Kuriyan, 2002; Nolen et al., 2004), that is highly conformationally variable and its conformation can influence both substrate binding and catalytic efficiency. The C-lobe serves as a docking site for substrate peptides/proteins. The N-terminal part of the peptide lies in a groove between the αD and αF-helices on one side and the αG-helix on the other side.

Bioinformatics approaches (Kornev et al., 2006) have shown that key hydrophobic residues in kinases have a strict organization around cores. In particular, the overall fold is joined by two hydrophobic non-contiguous motifs referred as “spines” (Kornev et al., 2008). The spines connect all critically important elements of the protein kinase molecule to a single hydrophobic helix (αF) that provides their positioning in space. These motifs regulate the protein kinase activity. The regulatory spine is typically assembled after phosphorylation loop and the catalytic one is completed by the ATP adenine ring. All these are anchored to the buried hydrophobic αF helix of the C lobe and provide the basic architecture of the core.

The “activation loop” is a highly flexible loop, which adopts an extended conformation in the active kinase domain to function as a binding platform for the peptide substrate. The experimental structures of kinases show that αC-helix (in the small lobe) and the activation loop (in the large lobe) adopt distinct conformations associated with the active or different inactive forms. This extended conformation is stabilized by phosphorylation, as observed in the active and phosphorylated kinase domain of the Src-family kinase Lck (Hantschel and Superti-Furga, 2004). In contrast to the active kinase, the inactive kinases are structurally diverse (Noble et al., 2004). This diversity arises because no catalytic requirements constrain the fold when it is inactive, with the presence of different inactive conformations that, nevertheless, share a number of common structural themes.

In many kinases αC-helix can rotate and change its position, thereby altering the orientation of key catalytic residues. In the active state of EGFR kinase, the activation loop maintains a β 9 strand and an overall conformation compatible with substrate binding while the αC-helix is adjacent to the ATP-binding site (the “αC-in”conformation), and the catalytic important Asp831 residue of the conserved “DFG” motif is found within that site (“DFG-in”). That is, in the active state, the phenylalanine (Phe) side-chain occupies the ATP-binding pocket, and the aspartate (Asp) side-chain is located in the outside of the pocket. When the so-called “DFG-flip” occurs, the Asp and Phe residues swap: the Asp side-chain rotates into the ATP-binding pocket, and the Phe side-chain rotates out of the ATP-binding pocket (DFG-out conformation), leading the kinases to the inactive state (Shan et al., 2009). Some human kinases were shown to be able to adopt the DFG-out conformation (Debondt et al., 1993; Sicheri et al., 1997; Xu et al., 1997) and it was suggested that the DFG-in and DFG-out conformations are in dynamic equilibrium and can interconvert to determine the kinase function (Levinson et al., 2006).

The protein kinase fold may switch between an active or inactive conformation by extra domains or separate subunits. How the active and inactive states are stabilized and how states interconvert are key questions in understanding kinase regulation. Typically, the activation segment contains a serine/threonine or tyrosine residue that can be phosphorylated. This site is referred to as first “phosphorylation site”; some kinases can have up to three phosphorylation site in the activation loop and perform function specific for these kinases. Other kinases, called non-RD kinases, lack the conserved arginine residue in kinase subdomain and it has been observed that some of them do not autophosphorylate the activation loop and are either constitutively active or regulated through alternative mechanisms (Dardick and Ronald, 2006). Most protein kinases are comprised of more than just the kinase domain. This can be flanked by other domains which tend to be quite dynamic or are part of a multi-subunit complex such as the Cyclin-dependent Kinases (CDK) which require an activating cyclin or phosphorylase kinase. Others such as the receptor tyrosine kinases are anchored to membranes and often have long segments that tether them to the membrane as well as long C-terminal tails. Certain protein kinases can be activated or inhibited by specific polypeptide cofactors. In CDK2 the activation depends on association with a cyclin subunit via the interaction of the αC-helix with specific hydrophobic residues on cyclin that enable the active site to be accessible for phosphorylation (Lowe et al., 1997; Lolli, 2010). Many protein kinases dimerize as part of their activation mechanism, and dimerization can be regarded as a special case of kinase activation by accessory proteins or domains. In such cases, either both partners are activated by reciprocal phosphorylation or one partner (the activator kinase) activates the other (the receiver kinase) through an allosteric mechanism (Endicott et al., 2012).

## Computational studies: an overview

Computational works focusing on kinase conformational transitions can be divided in two groups: those based on brute-force approach, where the system was simulated for microseconds and those where the dynamical evolution of the system was altered to speed up the sampling. Using the former approach and a special-purpose supercomputer, the group of D.E. Shaw described spontaneous transitions in the microsecond time-scale for the kinase domain of the EFGR from the active to the so-called “Src-like inactive” conformation by way of two sets of intermediate conformations, significantly different from the existing crystal structures. Interestingly, in one of them the helical arrangement of the activation loop (A-loop) leaves the well-known phosphorylation site exposed (Shan et al., 2013). Similarly, Yang et al. used several independent MD trajectories starting from the putative intermediate conformations to reconstruct the transitions pathways between active and inactive states for the Src kinase and identified the concerted conformational rearrangements in terms of structural regions of the protein, such as the A-loop and the αC-helix (Yang et al., 2009). They also found that transient structures in not fully active conformations allow exposure of Tyr416 to the bulk solvent—the residue whose phosphorylation locks the Src in the active state—a feature typical of the fully active states. In an extensive application of the Replica Exchange MD—an enhancing sampling technique favoring the overcome of potential energy barriers—Huang et al. found that the αC-helix acts as an energy switch between the active and inactive conformations and describe the sequence of events along the optimal inactivation path as the fold of the A-loop into the active site followed by the αC-helix movement (Huang et al., 2012).

Due to the large computational requirements, brute-force approaches are still limited to few research groups and inevitably to few systems. Therefore, a class of theoretical-computational methods able to sample the conformational rearrangements at limited computational costs were applied to protein kinases. Perturbative approaches, which are likely to be the most powerful techniques to model the free energy of a chemical reaction, are not particularly suited in this case, mainly because it is very difficult to find a proper reaction coordinate in a high-dimensional space able to fully discriminate the two conformational states. Three of such methods, the Targeted, Steered and Biased MD were applied to model the conformational transitions between the active/inactive forms of the catalytic domain of LYN kinase, a member of the Src family of protein tyrosine kinases: the results showed that although the transition pathways described by these approaches are similar, the path is strongly dependent on the choice of progress variable (Huang et al., 2009).

One of the most interesting approaches, however, is based on the possibility to iteratively select the conformations sampled by unbiased MD which are most likely to be productive, in order to limit the time spent in sampling the conformational space regions within the free-energy minimum basin. In this regard, Beckstein et al. applied the RMSD as a measure to apply their Dynamic IMportance Sampling (DIMS) algorithm to describe the zipping and unzipping of adenylate kinase (Beckstein et al., 2009). From their results, a cooperative salt bridge zipper is hypothesized to be the rate-limiting step of the apo-AdK conformational transition.

Following a similar approach, our group applied the Essential Dynamics algorithm to the description of the conformational transitions in the CDK2 (Bešker et al., 2013). The power of such a technique is due to the fact the direction in the high-dimensional space is described by the native protein movements in its stable conformational states, e.g., open and closed. In this case, the choice of the progress variable(s) is natural and self-defined by the unbiased MD simulations of the starting and target state. In the case of CDK2, we found that both the opening and closure follow common transition paths in the essential subspace, involving the same structural determinants (i.e., the αC-helix and the activation loop) but in opposite order, indicating that the time-sequence of such rearrangements determines the specific protein conformational transition. It is worth noting that due to their high computational efficiency, the last two methods can be used to fill the gap between single-system and system-wide studies, thus providing an unvaluable source of information at the protein family level.

All the above methods, with their advantages and shortcomings, represent an intriguing field of research and, in the case of the kinase conformational transitions, they have contributed to a better description of the transition pathways between the active and inactive forms.

Nevertheless, several important aspects from a biochemical point of view are still not well-characterized in detail, including for example the possibility to bridge the gap between the atomic description of the transitions and the protein activity and/or the role played by possible biological partners. In the next few years, with the support of new or refined enhancing sampling algorithm and the fast growth of the computational power, we envision that a better modeling of large conformational rearrangements of biomolecules, as those occurring in kinases, will be within range of the scientific community.

## Inhibitors

The central biological role of protein kinases leads to their frequent alteration in several pathologies, first of all cancer. It is not surprising, therefore, that they are major targets for therapy. The great number of family members and their high degree of conservation, however, easily lead to multi target inhibition and therefore important side effects. First-generation CDK inhibitors, for instance, targeted the non-specific ATP-binding site and were discarded in the pre-clinical phase because of their toxic effects (Lapenna and Giordano, 2009).

The importance of kinase structural flexibility on the rational drug design of their inhibitor can be appreciate looking at the experience of imatinib, the first Bcr-Abl inhibitor approved by the US Food and Drug Administration that revolutionized treatment of Chronic Myelogenous Leukaemia (CML). At the time of this drug discovery, the structural knowledge on the kinase family was not deep enough to rationalize its mode of action, but several subsequent studies demonstrated that imatinib exploits the change of conformation of the DFG-motif in the unphosphorylated inactive form (Lambert et al., 2013). These second generation inhibitors, targeting a variable region in the inactive form, are more capable of discerning among kinases but at the same time have the Achilles' heel of a greatest rate of drug-resistant mutations (Noble et al., 2004).

First generation inhibitors, in fact, targeting the active conformation, seldom developed drug-resistant mutations because they would have compromised the kinase activity. The war against cancer, therefore, moved another step, going against imatinib resistant cancers.

A growing number of experimentally solved kinase structures (more than 5000 in PDB database), both in active and inactive forms, and several MD simulations at microsecond-timescale have changed our knowledge on this protein family. These deep roots made possible the successful application of *in silico* rational drug design in a growing number of cases: (1) the so-called hybrid-design was carried out, in which the same kinase inhibitor targets different protein regions, thus merging the characteristic of potency (typical of first generation inhibitors) and selectivity (obtained by second generation ones) (Albaugh et al., 2012); (2) imatinib was also reengineered to reduce its cardiotoxicity, by making use of a different water propensity of two residues in the principal and secondary target kinases (Fernandez et al., 2007). Several MD simulations were carried out at this scope, that were able to discriminate the dynamic and structural characteristics of Bcr-Abl and C-Abl kinases, this last responsible for the cardiotoxic effects; (3) systematic rigid body docking of potential inhibitors against 84 unique protein kinases identified three derivatives of indirubin (Zahler et al., 2007), one of which has been recently indicated as particularly active against cancer metastasis (Braig et al., 2013).

The research on better kinase inhibitors by computational methods has focused not only on the target but on the ligand as well, with the so-called Pharmacophore Modeling approach, i.e., “an ensemble of steric and electronic features that is necessary to ensure the optimal supramolecular interactions with a specific biological target and to trigger (or block) its biological response” (Wermuth et al., 1998). Modern drug design combines more computational approach, as in the case of the identification of novel inhibitors against thymidine monophosphate kinase of *M. tuberculosis* with potent antitubercular activity, obtained through the combination of pharmacophore modeling, docking simulation, and structure interaction fingerprint analysis (Kumar et al., 2009).

It is easy to predict that these coming years will still be rich of new kinase inhibitors with improved selectivity and mutant resistance. In fact, new targets, coming from the computational and experimental characterization of kinase intermediates are combined with improved computer-aided drug design methods.

## Conclusions and perspectives

As briefly highlighted in this review, the intrinsic flexibility of the kinases represents a very intense field of research. The recent developments in theoretical-computational modeling are contributing to elucidate the actual dynamical behavior of the kinase domains, thus leading to the possibility of enhancing the design of new and selective inhibitors using a proper atomistic description of their conformational basins and the pathways along which they continuously move to tune their activity. Finally, the growth of computational power as well as the development of new theoretical treatments able to model slow and/or rare events at limited computational costs are making it possible to switch from single-system modeling of the kinase dynamical behavior to a system-wide modeling.

### Conflict of interest statement

The authors declare that the research was conducted in the absence of any commercial or financial relationships that could be construed as a potential conflict of interest.
